# Supplementary material for: Correlation between genetic and environmental risk factors for age-related macular degeneration in Brazilian patients
Source: PLoS One. 2022 Jun 3;17(6):e0268795. doi: 10.1371/journal.pone.0268795 (PMC9165864; doi:10.1371/journal.pone.0268795)
Supplement: S1 Fig — (DOCX) [file pone.0268795.s001.docx]

S1 FIG. PATIENTS ENVIRONMENTAL RISK FACTORS DATA CHART
