# Supplementary material for: Correlation between genetic and environmental risk factors for age-related macular degeneration in Brazilian patients
Source: PLoS One. 2022 Jun 3;17(6):e0268795. doi: 10.1371/journal.pone.0268795 (PMC9165864; doi:10.1371/journal.pone.0268795)
Supplement: S1 File — (DOCX) [file pone.0268795.s005.docx]

Questionnaire on environmental and genetic risk factors associated with age-related macular degeneration in a Brazilian population.

NAME:_________________________________DATE OF BIRTH:____/____/______

LOCAL OF BIRTH_______________________STATE__________________________

PROFESSION:________________________

SEX: □F □M

1. MAIN COMPLAINT(S):

□sudden visual impairment (VI) right eye (RE) □sudden visual impairment left eye (LE)

□ progressive VI RE □progressive VI LE

□central scotoma RE □central scotoma LE □paracentral scotoma RE □paracentral scotoma LE

□metamorphopsia RE □metmorphopsia LE

2. HISTORY OF AMD: □NO □YES

□mother □ father □brothers □siblings □grandparents □other(s) _______

3. SKIN COLOR -What race/ethnicity do you consider yourself?

□White □Mulatto/Brown □Black □Asian

4. IRIS COLOR

□light □medium □dark

4. USE OF GLASSES with UV or HAT: □ NO □ YES

5. REFRACTIVE ERROR(S) (>1D)

□NO □YES □myopia □hyperopia □astigmatism

6. SYSTEMIC DISEASES

□NO □YES □ systemic arterial hypertension □ (systolic >/= 160mmHg/diastolic >/=95 mmHg) □diabetes (fasting glycemia > 110mg/dL , insulin dependent) □diabetes (blood glucose > 110mg/dL, use of oral hypoglycemic agents) □angina □stroke episode □skin cancer □rheumatoid arthritis □thyroid disorders □arthrosis □other(s) ________

7. CATARACT: □NO □YES, RE □YES, LE

8. HIGH TOTAL CHOLESTEROL □NO YES □_______ HDL value_______________

9. HIGH TRIGLYCERIDES: □NO □YES _________

10. MEDICATION in USE (at least for 5 years)

□NO □YES □insulin □diuretic □betablocker □calcium blocker □aspirin □antiacid □anti-inflammatory □thyroid hormone □estrogen □progesterone □statin □oral hypoglycemic □other(s)____

11. VITAMINS/MINERALS

□NO □Centrum/Ocuvite/Vitergan/Vitalux □omega 3 □zinc □lutein/zeaxanthin for_______year(s) □other(s)__________________

12. FOOD CONSUMPTION (good intake of vegetables/fruits): □NO □YES

13. SMOKING (more than 100 cigarettes in life)

□NO □ YES ______cigarretes/day □former smoker □environmental smoker

14. SEDENTARISM (no physical activity at least 3times/week): □NO □YES

15. ALCOHOL CONSUMPTION: □NO □YES(> 3 daily doses) □former drinker

16. OPTICAL AID: □NO □YES:________________________

18. BODY MASS INDEX: □ <18.5 □18.5–24.9  □25–29.9  □>30

Questionnaire based on articles:

Tomany SC, Wang JJ, Van Leeuwen R, Klein R, Mitchell P, Vingerling JR, et al. Risk factors for incident age-related macular degeneration: pooled findings from 3 continents. Ophthalmology. 2004 jul;111(7):1280–7.

Chakravarthy U, Wong TY, Fletcher A, Piault E, Evans C, Zlateva G, et al. Clinical risk factors for age-related macular degeneration: a systematic review and meta-analysis. BMC Ophthalmol. 2010;10:31.

Tan JSL, Mitchell P, Smith W, Wang JJ. Cardiovascular risk factors and the long-term incidence of age-related macular degeneration: the Blue Mountains Eye Study. Ophthalmology. 2007 jun;114(6):1143–50
