# Supplementary material for: Correlation between genetic and environmental risk factors for age-related macular degeneration in Brazilian patients
Source: PLoS One. 2022 Jun 3;17(6):e0268795. doi: 10.1371/journal.pone.0268795 (PMC9165864; doi:10.1371/journal.pone.0268795)
Supplement: S2 File — (PDF) [file pone.0268795.s006.pdf]

FREE AND INFORMED CONSENT TERM

"STUDY OF RISK FACTORS ASSOCIATED WITH AGE-RELATED MACULAR DEGENERATION IN A BRAZILIAN POPULATION"

Responsible for the research: Dr. Priscila Hae Hyun Rim

Dear patient:

Age-related macular degeneration is a condition caused by hereditary and environmental factors, which can lead to low visual acuity in people over 50 years of age. Genetic components are often associated with the disease and may explain the observed eye disorders. We are inviting you to participate in a survey that may identify whether you have any genetic changes or predisposition to develop eye diseases associated with age-related macular degeneration. For this, a small amount of blood will be collected, today or if you prefer in the next appointment, to study whether or not genetic factors associated with this disease are present in your body.

Participation in this research is VOLUNTARY and your name (patient name) will be secret (through numerical coding) and anonymity; it will not appear in the search results, and there is therefore no possibility of being identified (maintaining total privacy). You (or patient under his or her legal responsibility) are not obliged to participate in this research and his/her treatment will be done in the same manner, including if he or she does not agree to participate.

The objective of this study is to determine which genes and environmental factors are most commonly involved in age-related macular degeneration and to provide information and guidance to patients and family members, indicating the best treatments and forms of prevention currently existing.

Any questions, please contact: Dr. Priscila Hae H. Rim - tel: (19) 3521-7380 Dr. Antonia Paula M. de Faria – tel: (19) 3521-7901

Research Ethics Committee: Rua Tessália Vieira de Camargo, 126 Cid. Universitaria Campinas - São Paulo-Brazil -areaCode:13083-970

Thank you for your attention.
