# Supplementary material for: Correlation between genetic and environmental risk factors for age-related macular degeneration in Brazilian patients
Source: PLoS One. 2022 Jun 3;17(6):e0268795. doi: 10.1371/journal.pone.0268795 (PMC9165864; doi:10.1371/journal.pone.0268795)
Supplement: Supplementary file 5 [file pone.0268795.s003.docx]

PATIENTS #	LOC	CFH	HTRA1
1	GG	TT	AG
2	GG	TC	GG
3	GT	TT	AG
4	GT	TC	AA
5	GT	CC	AA
6	GT	TT	AA
7	TT	TT	AG
8	GT	CC	AA
9	GG	CC	AG
10	TT	TT	GG
11	TT	CC	AA
12	GG	CC	AA
13	GG	TC	AG
14	TT	TC	AA
15	GG	TC	AG
16	GG	TC	AG
17	GG	CC	AA
18	GT	TC	AG
19	GT	TC	AA
20	TT	TT	AA
21	GT	TT	AG
22	GT	TC	AA
23	GT	TC	AG
24	GG	TC	GG
25	TT	TC	AA
26	GT	CC	AA
27	GT	TT	AG
28	TT	TT	AA
29	GG	TT	GG
30	GT	CC	AG
31	GT	TC	GG
32	TT	TT	AA
33	GT	CC	AG
34	TT	CC	AA
35	GT	TC	AA
36	TT	CC	AG
37	GT	TT	AG
38	GT	TC	AA
39	TT	TT	AA
40	GT	TT	AG
41	TT	CC	AA
42	GG	TT	GG
43	GT	CC	AG
44	TT	TC	AA
45	GT	TC	GG
46	GG	CC	AG
47	GT	TT	AG
48	GT	CC	GG
49	GG	TT	GG
50	GG	TT	AG
51	GG	TC	AA
52	TT	TC	AA
53	GG	TC	AA
54	GT	TT	AA
55	GT	TC	AG
56	GT	CC	GG
57	GG	TT	AA
58	GG	TC	AG
59	GG	CC	GG
60	GG	TC	AG
61	GT	TC	AA
62	GG	CC	AG
63	GG	TC	AA
64	GG	TC	AA
65	TT	TC	AA
66	GG	TC	AG
67	GG	TT	GG
68	GT	TT	AA
69	TT	TT	AA
70	GG	TC	AG
71	TT	TT	AG
72	GG	TC	AA
73	GG	CC	AG
74	GT	TC	AA
75	GG	TC	GG
76	GG	TT	AG
77	GG	TT	AA
78	GG	TC	AG
79	GG	TC	AA
80	GT	TC	AG
81	GT	TC	AG
82	GT	CC	AG
83	GG	CC	AG
84	GG	TT	AG
85	GT	TC	AA
86	GG	TC	AG
87	GT	TC	AA
88	GT	TC	AG
89	TT	CC	AA
90	GT	TC	AG
91	GG	TC	AG
92	GG	TC	AG
93	TT	TC	AA
94	GG	TT	GG
95	GT	TT	AG
96	GG	TC	AG
97	GT	TT	GG
98	TT	TC	AG
99	GG	TC	AG
100	GT	TT	AA
101	GT	TC	AG
102	TT	TT	AG
103	GT	TT	AA
104	GG	TC	AA
105	GT	TC	GG
106	TT	TT	AA
107	GG	CC	AG
108	GT	CC	AA
109	GG	TC	AG
110	GG	TC	AG
111	GG	TC	AA
112	GT	TT	AA
113	GT	TC	AA
114	TT	TC	AG
115	GT	TC	AA
116	GT	TC	AG
117	GT	TC	AG
118	GT	TT	AG
119	TT	TC	AA
120	GG	TC	AG
121	GT	TC	AA
122	GT	TC	AA
123	GT	CC	GG
124	GT	TC	AG
125	GG	CC	AG
126	TT	TC	AG
127	GG	CC	AG
128	GT	CC	GG
129	GT	CC	AG
130	TT	TT	AA
131	GG	TT	GG
132	GG	TT	AA
133	GT	TC	AG
134	GG	TC	AG
135	GG	TC	GG
136	GG	TT	AG
137	GT	TC	AG
138	GT	TC	AA
139	GT	TC	AG
140	GT	TC	AG
141	GT	TC	AG

ABBREVIATIONS OF VARIANTS OF GENE

LOC387715 (rs10490924)
CFH (rs1061170)
HTRA1 (rs11200638)
