# Supplementary material for: Correlation between genetic and environmental risk factors for age-related macular degeneration in Brazilian patients
Source: PLoS One. 2022 Jun 3;17(6):e0268795. doi: 10.1371/journal.pone.0268795 (PMC9165864; doi:10.1371/journal.pone.0268795)
Supplement: Supplementary file 6 [file pone.0268795.s004.docx]

CONTROLS #	LOC	CFH	HTRA1
1	GG	TC	GG
2	GG	TT	AG
3	GG	TC	AG
4	GG	TT	AA
5	GT	TC	AA
6	GT	TC	AG
7	GT	TT	AA
8	GT	TC	AG
9	GG	CC	AG
10	GG	TC	GG
11	GG	TC	GG
12	GG	TC	AA
13	GT	TT	GG
14	GG	CC	AG
15	GT	TT	AG
16	GT	CC	AG
17	GT	TT	AA
18	GT	TT	AG
19	GT	TT	GG
20	GG	TC	GG
21	GG	TT	AG
22	GG	TC	AG
23	GG	TT	GG
24	GT	TC	GG
25	GG	TT	AA
26	GT	TT	GG
27	GT	TT	AG
28	GG	TT	AA
29	GG	TC	AG
30	GT	TT	AG
31	GT	TC	AG
32	GG	CC	GG
33	GT	TT	AG
34	GT	TT	AG
35	GG	TT	AG
36	GG	TC	AG
37	GG	TC	GG
38	GG	TC	AG
39	GG	TC	AG
40	GT	TT	AG
41	GT	TC	AG
42	GT	TT	GG
43	GG	TT	AG
44	GT	TC	AG
45	GG	CC	AG
46	GG	TT	GG
47	GG	TT	AG
48	GT	TC	GG
49	GG	TT	AG
50	GT	TC	AG
51	GG	TC	AG
52	GT	TC	GG
53	GG	TT	AG
54	GT	TT	AG
55	GT	TT	GG
56	TT	TC	GG
57	GT	TT	AG
58	GG	TT	AG
59	GG	TC	AG
60	GT	TT	GG
61	GG	TT	AA
62	GG	TC	AG
63	GT	TT	GG
64	GG	TC	AA
65	GG	TT	AG
66	GG	TC	AA
67	GT	TT	GG
68	GT	TT	AG
69	GG	TT	GG
70	GG	TC	AA
71	GG	TT	GG
72	GG	TC	GG
73	GG	TT	GG
74	GT	TC	GG
75	GT	TT	AG
76	GG	TT	GG
77	GG	TT	AG
78	GG	TT	AG
79	GG	TC	GG
80	GG	TC	GG
81	GT	TC	AG
82	GT	TT	AG
83	GT	TT	GG
84	GT	TC	AG
85	GG	CC	GG
86	GG	TC	GG
87	GG	TT	AG
88	GG	TC	AG
89	GT	TC	GG
90	GT	TT	AG
91	GG	TT	AG
92	GG	TC	AG
93	GG	TT	AG
94	TT	TT	AG
95	GT	TC	GG
**ABBREVIATIONS OF VARIANTS OF GENES**			
LOC387715 (rs10490924)			
CFH (rs1061170)			
HTRA1 (rs11200638)			
