# Supplementary material for: Correlation between genetic and environmental risk factors for age-related macular degeneration in Brazilian patients
Source: PLoS One. 2022 Jun 3;17(6):e0268795. doi: 10.1371/journal.pone.0268795 (PMC9165864; doi:10.1371/journal.pone.0268795)
Supplement: Attachment — Submitted filename: RESPONSE TO REVIEWER PLOS .docx [file pone.0268795.s007.docx]

PONE-D-21-33908

CORRELATION BETWEEN GENETIC AND ENVIRONMENTAL RISK FACTORS FOR AGE-RELATED MACULAR DEGENERATION IN BRAZILIAN PATIENTS

PLOS ONE

EDITOR COMMENT:

*Thank you for submitting your manuscript to PLOS ONE. After careful consideration, we feel that it has merit but does not fully meet PLOS ONE’s publication criteria as it currently stands. Therefore, we invite you to submit a revised version of the manuscript that addresses the points raised during the review process.*

*The expert reviewer has raised questions about your classification of AMD cases, about the questionnaire that you used, and about the diversity of the Brazilian population. Please address these concerns. Although importance is not a criterion for publication, it would be helpful if you could address the novelty of your research.*

RESPONSE: Thank you for considering our manuscript to review process. We have revised the manuscript and addressed Reviewer’s comments.

**Journal Requirements:**

When submitting your revision, we need you to address these additional requirements.

1. Please ensure that your manuscript meets PLOS ONE's style requirements, including those for file naming.

RESPONSE: We have checked and attest that all formatting and style requirements have been met.

2. Please ensure that you have specified (1) whether consent was informed, (2) what type you obtained (for instance, written or verbal, and if verbal, how it was documented and witnessed). If your study included minors, state whether you obtained consent from parents or guardians. If the need for consent was waived by the ethics committee and (3) If you are reporting a retrospective study of medical records or archived samples, please ensure that you have discussed whether all data were fully anonymized before you accessed them and/or whether the IRB or ethics committee waived the requirement for informed consent. If patients provided informed written consent to have data from their medical records used in research, please include this information.

**RESPONSE**: **We** performed **a** cross-sectional study with a control group and we ensure that the Ethics Committee the University of Campinas (www.fcm.unicamp/pesquisa/etica/index.html) approved this study under protocol #881/2007, and all participants provided **written** informed consent through the following model:

FREE AND INFORMED CONSENT TERM

**"STUDY OF RISK FACTORS ASSOCIATED WITH AGE-RELATED MACULAR DEGENERATION IN A BRAZILIAN POPULATION"**

Responsible for the research: Dr. Priscila Hae Hyun Rim

Dear patient:

Age-related macular degeneration is a condition caused by hereditary and environmental factors, which can lead to low visual acuity in people over 50 years of age. Genetic components are often associated with the disease and may explain the observed eye disorders. We are inviting you to participate in a survey that may identify whether you have any genetic changes or predisposition to develop eye diseases associated with age-related macular degeneration. For this, a small amount of blood will be collected, today or if you prefer in the next appointment, to study whether or not genetic factors associated with this disease are present in your body.

Participation in this research is VOLUNTARY and your name (patient name) will be secret (through numerical coding) and anonymity; it will not appear in the search results, and there is therefore no possibility of being identified (maintaining total privacy). You (or patient under his or her legal responsibility) are not obliged to participate in this research and his/her treatment will be done in the same manner, including if he or she does not agree to participate.

The objective of this study is to determine which genes and environmental factors are most commonly involved in age-related macular degeneration and to provide information and guidance to patients and family members, indicating the best treatments and forms of prevention currently existing.

Any questions, please contact: Dr. Priscila Hae H. Rim - tel: (19) 3521-7380 Dr. Antonia Paula M. de Faria – tel: (19) 3521-7901

Research Ethics Committee: Rua Tessália Vieira de Camargo, 126 Cid. Universitaria Campinas - São Paulo-Brazil -areaCode:13083-970

Thank you for your attention.

**RESPONSE TO REVIEWER 1:**

REVIEWER COMMENT: I believe the study is well performed and clearly written. However, there are a few issues that called my attention.
1. The classification and definition of AMD shoud be reviewed. Authors classisfy the disease in early and advance, (early including early and intermediate AMD). I believe this is not accurate and can lead to confussion, it would be better to classify patients into early, intermediate and advanced AMD. If this is not possible, then at least the term early should be changed to "not-advanced" AMD.
Further, authors should specify if the clinical features listed as diagnostic for wAMD and geographic atrophy were based on clinical fundoscopy, fundus photography, OCT or other methods. Moreover, some of the clinical characteristics listed to define wAMD are not specific for the disease (RPE detachment, epiretinal membrane, hard exudates, phocoagulation scars) while others are missing (intraretinal or subretinal fluid). The same is true for the definition of geographic atrophy (Ex: hypopigmentation does not imply the presence of atrophy). Why is an area of 175microns used to determine atrophy? A deep review to the inclusion and exlcusion criteria should be done and better explained.

RESPONSE: We thank the reviewer for this observation. We agree that the observations of the reviewer are exact and need modifications.

The definition of wet AMD were modified according to your observations. We applied the definition of geographic atrophy according to THE INTERNATIONAL CLASSIFICATION AND GRADING SYSTEM FOR AGE-RELATED MACULOPATHY AND AGE-RELATED MACULAR DEGENERATION, “geographic atrophy is graded according to presence, location, and area affected. The minimum GA area size is that of the circle corresponding to 175-mm diameter”; nevertheless we assure that the inclusion of patients with GA were accurate.

Bird AEC, Bressler NM, Bressler SB, et al: An International Classification and Grading System for Age- Related Macul- 32. opathy and Age-Related Macular Degeneration Surv Oph- thalmol 39:367--74, 1995

The following modifications have been added:

We assure that all subjects underwent a complete ophthalmological examination, including biomicroscopy, retinography, fundoscopy, fluorescein angiography and optical coherence tomography (OCT).

Wet AMD was defined as the presence of at least one of the established characteristics (sub-retinal or sub-RPE neovascular membranes; intraretinal, sub-retinal, or sub-epithelial pigmented scar or glial tissue; intra- or sub-retinal or sub-RPE fluid or hemorrhage). Dry AMD was defined as a geographic atrophy of the RPE with either delimited zone of partial or complete depigmentation or apparent lack of RPE, in which the choroidal vessels of the eye are visible in an area larger than 175 μm. Wet AMD and GA involving center of macula were considered to be advanced forms of AMD.

REVIEWER COMMENT 2. It would be very helpful to know which questionaire was used to assess demographic and general health characteristics. Is it a validated questionaire, is it one proposed by the authors (if so, further ditails should be incuded).

RESPONSE: We proposed our own questionnaire based on articles listed below. Among more than 70 risk factors described in literature, the most frequently associated with AMD and easily identifiable in clinical practice were highlighted in this study.

Tomany SC, Wang JJ, Van Leeuwen R, Klein R, Mitchell P, Vingerling JR, et al. Risk factors for incident age-related macular degeneration: pooled findings from 3 continents. Ophthalmology. 2004 jul;111(7):1280–7.

Chakravarthy U, Wong TY, Fletcher A, Piault E, Evans C, Zlateva G, et al. Clinical risk factors for age-related macular degeneration: a systematic review and meta-analysis. BMC Ophthalmol. 2010;10:31.

Tan JSL, Mitchell P, Smith W, Wang JJ. Cardiovascular risk factors and the long-term incidence of age-related macular degeneration: the Blue Mountains Eye Study. Ophthalmology. 2007 jun;114(6):1143–50

COPY OF THE APPLIED QUESTIONNAIRE:

Questionnaire on environmental and genetic risk factors associated with age-related macular degeneration in a Brazilian population.

NAME:_________________________________DATE OF BIRTH:____/____/______

LOCAL OF BIRTH_______________________STATE__________________________

PROFESSION:________________________

SEX: □F □M

1. MAIN COMPLAINT(S):

□sudden visual impairment (VI) right eye (RE)	□sudden VI left eye (LE)

□ progressive VI RE	□progressive VI LE

□central scotoma RE □central scotoma LE	□paracentral scotoma RE	□paracentral scotoma LE

□metamorphopsia RE □metmorphopsia LE

2. HISTORY OF AMD: □NO	□YES

□mother □ father □brothers	□siblings	□grandparents □other(s) _______

3. SKIN COLOR -What race/ethnicity do you consider yourself?

□White □Mulatto/Brown	□Black	□Asian

4. IRIS COLOR

□light	□medium	□dark

4. USE OF GLASSES with UV or HAT: □ NO □ YES

5. REFRACTIVE ERROR(S) (>1D)

□NO	□YES □myopia	□hyperopia □astigmatism

6. SYSTEMIC DISEASES

□NO	□YES	□ systemic arterial hypertension □ (systolic >/= 160mmHg/diastolic >/=95 mmHg) □diabetes (fasting glycemia > 110mg/dL , insulin dependent)	□diabetes (blood glucose > 110mg/dL, use of oral hypoglycemic agents)	□angina	□stroke episode □skin cancer □rheumatoid arthritis	□thyroid disorders	□arthrosis	□other(s) ________

7. CATARACT: □NO □YES, RE □YES, LE

8. HIGH TOTAL CHOLESTEROL □NO	YES □_______ HDL value_______________

9. HIGH TRIGLYCERIDES: □NO □YES _________

10. MEDICATION in USE (at least for 5 years)

□NO □YES □insulin □diuretic □betablocker □calcium blocker □aspirin □antiacid □anti-inflammatory □thyroid hormone □estrogen □progesterone □statin □oral hypoglycemic □other(s)____

11. VITAMINS/MINERALS

□NO □Centrum/Ocuvite/Vitergan/Vitalux □omega 3	□zinc	□lutein/zeaxanthin for_______year(s) □other(s)__________________

12. EATING HABITS (good intake of vegetables/fruits at least 3 times/week): □NO □YES

13. SMOKING (more than 100 cigarettes in life)

□NO □ YES ______cigarretes/day □former smoker □environmental smoker

14. SEDENTARISM (no physical activity at least 3 times/week): □NO □YES

15. ALCOHOL CONSUMPTION: □NO □YES(> 3 daily doses) □former drinker

16. OPTICAL AID: □NO □YES:________________________

18. BODY MASS INDEX: □ <18.5 □18.5–24.9  □25–29.9  □>30

REVIEWER COMMENT 3. Brazil has a very diverse population, something very important when performing genetic studies. How was this addressed? We patients from different ethnicities included?

RESPONSE: AMD in general occurs earlier and more frequently in Caucasians than in Asians and black Americans according to findings in literature meanwhile conflicting results between studies and in relation to advanced forms of AMD there was no significant difference. In our study, there was no significant association of skin color between the groups of AMD patients and controls and when comparing types of AMD (please see tables below).

Demographic and clinical characteristics of the AMD cases and controls

| AMD cases (n=141) | Controls  (n=95) | | | χ^2^ | | p |  |
| --- | --- | --- | --- | --- | --- | --- | --- |
| Skin colour  White  Brown  Black | | 94(66.7%)  43(30.5%)  4(2.8%) | 62(65.3%)  29(30.5%)  4(4.2%) | | 0.333 | 0.847 | |

Demographic and clinical characteristics of the AMD cases according to AMD status

| Variables | Not-advanced  AMD  (n=42) | Advanced AMD  (n=99) | χ^2^ | p |
| --- | --- | --- | --- | --- |

| Skin colour  White  Brown  Black | 25(59.5%)  15(35.7%)  2(4.8%) | 69(69.7%)  28(28.3%)  2(2%) | 1.773 | 0.412 |
| --- | --- | --- | --- | --- |

Racial Differences in Age-Related Macular Degeneration Rates in the United States: A Longitudinal Analysis of a Managed Care Network Brian L. VanderBeek1, David N. Zacks1, Nidhi Talwar1, Bin Nan2, David C. Musch1,3, and Joshua D. Stein1 1Department of Opht Am J Ophthalmol. 2011 August ; 152(2): 273–282.e3. doi:10.1016/j.ajo.2011.02.004.

Liutkeviciene R, Lesauskaite V, Asmoniene V, Zaliūniene D, Jasinskas V. Factors determining age-related macular degeneration: a current view. Medicina (Kaunas). 2010;46(2):89–94.

Klein R, Klein BEK, Knudtson MD, Wong TY, Cotch MF, Liu K, et al. Prevalence of age-related macular degeneration in 4 racial/ethnic groups in the multi-ethnic study of atherosclerosis. Ophthalmology. 2006 mar;113(3):373–80.

REVIEWER COMMENT 4.Methods sections would be clearer if devided in subheadings

RESPONSE: We thank the reviewer for this observation; the following modifications have been added:

METHODS

**Subjects**

A cross-sectional study with control group was performed in 236 participants aged 50 years or older with 141 individuals with age-related macular degeneration and 95 controls without disease, all current patients from the Department of Ophthalmology-Otorhinolaryngology of Clinical Hospital, Faculty of Medical Sciences – UNICAMP. Written informed consent was obtained from all participants. The research protocol was in keeping with the tenets of the Declaration of Helsinki and Research Ethics Committee approvals were obtained.

**Questionnaire and clinical evaluation**

All subjects were interviewed by the researcher and completed a questionnaire consisting of “closed answers” (multiple choice) including questions about demographic factors, ocular and medical history, family history of AMD, lifestyle and smoking and drinking habits. Ocular examinations were performed including visual acuity, slit lamp evaluation and formal grading of colour stereoscopic fundus photographs(47) in all participants.

**Age-related macular degeneration grading**

The Age-Related Eye Disease Study (AREDS) grading system for AMD was used to classify the type of disease(48). Absence of disease or category 1 was defined as the presence of <10 drusen of < 63µm size. Early AMD/category 2 was defined as the presence of ≥10 drusen (<63µm size) or < 20 intermediate drusen (63 - 125µm size) or presence of hyper/hypo pigmentation; intermediate AMD/category 3 as the presence of ≥20 intermediate drusen or any large drusen (≥125µm size), and advanced or late AMD/category 4 by the presence of either geographic atrophy or choroidal neovascularization. Signs of neovascular or exudative AMD were RPE detachment or serous detachment of the sensory retina, subretinal or sub-RPE hemorrhages, and subretinal fibrous scars. Geographic atrophy was defined as a circular area (≥175 µm size) of retinal depigmentation with visible choroidal vessel, in the absence of neovascular AMD.

**Control group**

The control group consisted of patients older than 50 years of age who presented no evidence of AMD, such as drusen or changes in the RPE. These individuals consisted of non-family companions of patients examined in the retina clinic, as well as patients examined in other subspeciality wards of the local ophthalmology department. Exclusion criteria consisted of high myopia, angioid streaks, infective or inflammatory chorioretinal disease, or trauma. All subjects received a complete ophthalmic examination, including visual acuity measurement, refraction, biomicroscopy, and fundoscopy. Fluorescein angiography was performed in patients with wet AMD, and potential subjects with suspected neovascularization from retinal angiomatous proliferation or polypoidal choroidal vasculopathy were also excluded.

**Genotyping**

DNA was extracted from leukocytes present in 4 mL to 8 mL of peripheral blood. The *LOC387715 (rs10490924)*, *CFH (rs1061170)*, and *HTRA1 (rs11200638)* variants were evaluated using PCR and enzymatic digestion or PCR and direct sequencing, as in previous studies (29)(30)(31).

**Statistical analysis**

Data were entered into database using Microsoft Access-98. The SSPS 15.0 program was used for tabulations and statistical analysis. The findings in the index cases were compared with a control group with no disease. The reported statistical inferences are based on chi-square (χ^2^) analysis and the two sample Mann-Whitney tests for comparing means with the two-sided p value 0.05. Age, visual acuity in LogMAR and BMI were fitted as continuous variables. Logistic regression was performed using each AMD lesion as a dependent variable.

REVIEWER COMMENT 5.It is not clear the novelty offered by the results of this manuscript? what makes it special or different from previous published works? This should be explained in the discussion sections, otherwise, it just seems like a confirmation of things which are already known.

RESPONSE: We thank the reviewer for this observation. This study on risk factors of AMD in Brazilian population named AMD PROJECT has been conducted since 2008 when our group was formed. For the first time in Brazil we determined the main risk factors to the development of this condition and we aimed to analyze the correlation between the genetic and environmental factors and its implication on preventive measures. In a developing country like Brazil, the burden of treatment is high and impractical in many cases due to the cost and lack of assessment to health care system frequently leading to blindness.

The differential of this study is that for this highly admixed population even though genetic factors were similar to data worldwide, we found that environmental factors like cardiovascular, sedentarism and high blood cholesterol were significative in the development of AMD. Other point is that the correlational analysis of environmental and genetic factors allows us to conclude that the combined weight of the variables as number of *HTRA1* and *CFH* alleles, family history, number of LOC alleles, CVD, in the determination of AMD, is 25.6%, a result with statistical significance. These findings would contribute to target the preventive measures including a strong campaign on prevention of blindness promoted by the public health systems though action over potentially modifiable lifestyle factors and impact on AMD outcomes (already mentioned in the discussion section)

The following paragraph has been added in the discussion section to complete this point:

Determining the status of Brazilian patients with this heterogeneous disease and inherent peculiar behavioral, clinical, and genetic risk factors will contribute to guide clinical management decisions and impact on AMD outcomes.
